# Post-Treatment with Amorfrutin B Evokes PPARγ-Mediated Neuroprotection against Hypoxia and Ischemia

**DOI:** 10.3390/biomedicines9080854

**Published:** 2021-07-21

**Authors:** Agnieszka Wnuk, Karolina Przepiórska, Bernadeta A. Pietrzak, Małgorzata Kajta

**Affiliations:** Laboratory of Neuropharmacology and Epigenetics, Department of Pharmacology, Maj Institute of Pharmacology, Smetna Street 12, 31-343 Krakow, Poland; wnuk@if-pan.krakow.pl (A.W.); przepior@if-pan.krakow.pl (K.P.); pietrzak@if-pan.krakow.pl (B.A.P.)

**Keywords:** hypoxia, ischemia, stroke, perinatal asphyxia, neuroprotection, selective PPARγ modulator, post-treatment

## Abstract

In this study, we demonstrate for the first time that amorfrutin B, a selective modulator of peroxisome proliferator-activated receptor gamma—PPARγ, can protect brain neurons from hypoxia- and ischemia-induced degeneration when applied at 6 h post-treatment in primary cultures. The neuroprotective effect of amorfrutin B suggests that it promotes mitochondrial integrity and is capable of inhibiting reactive oxygen species—ROS activity and ROS-mediated DNA damage. PPARγ antagonist and *Pparg* mRNA silencing abolished the neuroprotective effect of amorfrutin B, which points to agonistic action of the compound on the respective receptor. Interestingly, amorfrutin B stimulated the methylation of the *Pparg* gene, both during hypoxia and ischemia. Amorfrutin B also increased the protein level of PPARγ during hypoxia but decreased the mRNA and protein levels of PPARγ during ischemia. Under ischemic conditions, amorfrutin B-evoked hypermethylation of the *Pparg* gene is in line with the decrease in the mRNA and protein expression of PPARγ. However, under hypoxic conditions, amorfrutin B-dependent hypermethylation of the *Pparg* gene does not explain the amorfrutin B-dependent increase in receptor protein expression, which suggests other regulatory mechanisms. Other epigenetic parameters, such as HAT and/or sirtuins activities, were affected by amorfrutin B under hypoxic and ischemic conditions. These properties position the compound among the most promising anti-stroke and wide-window therapeutics.

## 1. Introduction

Cerebrovascular accidents (commonly known as strokes) are the second leading cause of death and the leading cause of disability worldwide. Patients who survive stroke incidents experience loss of vision and/or speech, paralysis, and confusion [1]. The only pharmacological treatment approved by the Food and Drug Administration for acute ischemic stroke is recombinant tissue plasminogen activator (rt-PA), which is effective only if administered until 4.5 h after stroke onset. Moreover, rt-PA has a long list of contraindications, such as excitotoxicity, hemorrhage, and cerebral edema, and for this reason, only 5% of ischemic patients can be treated with rt-PA [2,3]. Additional stroke treatment is surgical thrombectomy, which can be used only up to 8 h following the onset of symptoms located in the anterior circulation and is eligible for no more than 2% of patients [4]. 

Aside from the stroke, another issue is perinatal asphyxia. Oxygen deprivation is considered the most common cause of death in fetuses and newborns, i.e., 2–4 newborns die per 1000 births. Each year, one million children die due to hypoxia and neonatal oxygen deprivation, which leads to permanent brain damage and disability such as hypoxic-ischemic encephalopathy [5]. Oxygen therapy and moderate hypothermia occurring up to 6 h after the hypoxic episode are the gold standard treatment for neonatal asphyxia [6].

The sudden death of brain cells caused by stroke and perinatal asphyxia results from a lack of oxygen (hypoxia) and/or glucose (ischemia). In the center of the damage (core), cell death occurs as a result of rapidly progressing necrosis. However, the cells surrounding the core of injury (the transition zone or ischemic penumbra) are damaged much slower (from 6 h to 3 days). For that reason, these cells are the main goals of neuroprotection.

During stroke and perinatal asphyxia, oxidative stress is strongly associated with brain injury. Hypoxia and ischemia incidents increase reactive oxygen species (ROS) production arising from an imbalance between prooxidants and antioxidants and overwhelm the antioxidant defenses. ROS generation can damage cell structures, including lipids, membranes, proteins, and DNA, leading to different types of cell death, including autophagy, apoptosis, and necrosis. Moreover, during the restoration of blood flow (also known as reperfusion), brain cell oxygenation increases, leading to a second burst of ROS generation.

Peroxisome proliferator-activated receptor gamma (PPARγ) belongs to a subfamily of the nuclear receptor superfamily as a ligand-activated transcription factor. When bound to ligands (such as unsaturated fatty acids, eicosnanoids, oxidized phospholipids), PPARγ dimerizes with the retinoid X receptor (RXR). As a heterodimer, it activates various genes, including those involved in cell proliferation, differentiation, metabolism, and inflammatory responses of the central nervous system [7,8]. Full PPARγ agonists are thiazolidinediones (TZDs), which are commonly used antidiabetic drugs, including pioglitazone and rosiglitazone. The role of TZDs in diabetes is to increase insulin sensitivity and improve glycemic control. Treatment with TZDs has also been shown to be neuroprotective in ischemic stroke, Alzheimer’s disease, and Parkinson’s disease [9,10]. Nevertheless, preclinical and clinical studies have shown that TZDs cause severe side effects such as bone loss, weight gain, and fluid retention, which can exacerbate congestive heart failure and highly limit their therapeutic potential [11].

Selective PPARγ modulators (SPPARγMs), which transactivate the expression of PPARγ-dependent reporter genes as partial agonists, could be safer alternatives to PPARγ full agonists [12]. Amorfrutin B is a plant-derived compound that belongs to SPPARγMs first isolated from the edible parts of legumes *Glycyrrhiza foetida* and *Amorpha fruticosa* [13]. Amorfrutin B has a PPARγ-binding affinity similar to that of the standard PPARγ-targeting drug rosiglitazone, and it can cross the blood–brain barrier and accumulate in brain tissue [14,15]. Unlike TZDs, amorfrutin B treatment showed liver-protecting properties, did not induce weight gain, and had no adverse effects on osteoblastogenesis or fluid retention. Nevertheless, little is known about the neuroprotective capacity of amorfrutin B.

As amorfrutin B can selectively activate PPARγ without evoking adverse effects known for TZDs, in the current study, we aimed to assess the neuroprotective properties of amorfrutin B in a cellular model of stroke and perinatal asphyxia. We hypothesize that targeting PPARγ with amorfrutin B causes a neuroprotective effect when administered post-treatment in experimental models of stroke and perinatal asphyxia in vitro.

## 2. Materials and Methods

### 2.1. Materials

The phosphate buffered saline (PBS) was from BIOMED LUBLIN (Lublin, Poland). The DNA/RNA Oxidative Damage (High Sensitivity) ELISA Kit was purchased from Cayman Chemical (Ann Arbor, MI, USA). The rabbit polyclonal anti-HIF1α antibody (3716) was purchased from Cell Signaling Technology (Danvers, MA, USA). B27 and neurobasal media were obtained from Gibco (Grand Island, NY, USA). Bradford reagent, sodium dodecyl sulfate, 30% acrylamide, 0.5 M Tris-HCl buffer, 1.5 M Tris-HCl gel buffer, Laemmli sample buffer and sodium dodecyl sulfate (SDS) were obtained from Bio-Rad Laboratories (Munchen, Germany). 2-Mercaptoethanol was purchased from Carl Roth GmbH + Co. KG (Karlsruhe, Germany). Immobilon-P membranes were purchased from Millipore (Bedford, MA, USA). The ROS-Glo™ H2O2 assay was obtained from Promega (Madison, WI, USA). The cytotoxicity detection kit and BM chemiluminescence Western blotting substrate (POD) were purchased from Roche Diagnostics GmbH (Mannheim, Germany). ELISA kits for PPARγ, PGC1α, and ADIPOQ were purchased from Shanghai Sunred Biological Technology Co. (Sunred, China). The culture plates were obtained from TPP Techno Plastic Products AG (Trasadingen, Switzerland). The mouse monoclonal anti-PPARγ antibody (sc-7273), rabbit polyclonal anti-MAP2 antibody (sc-20172), mouse monoclonal anti-β-actin antibody (sc-47778), and *Pparγ* siRNA were purchased from Santa Cruz Biotechnology, Inc. (Santa Cruz, CA, USA). Amorfrutin B, 2-Chloro-5-nitro-N-phenylbenzamide (GW9662), L-glutamine, fetal bovine serum (FBS), dimethyl sulfoxide (DMSO), 4-(2-hydroxyethyl)-1-piperazineethanesulfonic acid (HEPES), 3-[(3-Cholamidopropyl)dimethylammonio]-1-propanesulfonate hydrate (CHAPS), ammonium persulfate, N,N,N′,N′-tetramethylethane-1,2-diamine (TEMED), 2-Amino-2-(hydroxymethyl)-1,3-propanediol (TRIZMA base), Tween 20, DL-dithiothreitol, sodium deoxycholate, protease inhibitor (ethylenediaminetetraacetic acid-free), bromophenol blue, 2′,7′-dichlorofluorescein diacetate, radioimmunoprecipitation assay buffer (RIPA) buffer, Fluoro-Jade C, thiazolyl blue tetrazolium bromide, imprint methylated DNA quantification kits, sirtuin activity assay kits, histone deacetylase assay kits, histone acetyltransferase activity assay kits, protease inhibitor cocktail for mammalian tissues, and polyornithine were obtained from Sigma-Aldrich (St. Louis, MO, USA). AllStars Negative Control siRNA AF 488 and the RNeasy Mini Kit were obtained from Qiagen (Hilden, Germany). INTERFERin was obtained from PolyPlus Transfection (Illkirch, France), and the anti-PGC1α antibody (PA5-38021), Alexa Fluor Plus 488-conjugated anti-mouse IgG (A32723), Alexa Fluor Plus 647-conjugated anti-rabbit IgG (A32733), High Capacity cDNA-Reverse Transcription Kit, the TaqMan Gene Expression Master Mix, and TaqMan probes for specific genes encoding *Hprt*, *Hif1a*, *Pparg*, *Pgc1a*, and *Adipoq* were obtained from Thermo Fisher Scientific (Waltham, MA, USA). Quick-gDNA™ MicroPrep was obtained from ZymoResearch (Irvine, CA, USA).

#### 2.1.1. Primary Neuronal Cell Cultures

Neocortical tissues for neuronal cell cultures were obtained from Swiss CD1 mouse strain embryos (Charles River, Germany) at 15 days of gestation and cultured as described previously [16]. Briefly, neuronal cells were seeded on polyornithine-coated multiwell dishes at a density of 2.5 × 10^5^ cells per cm^2^ and cultivated in phenol red-free neurobasal medium containing fetal bovine serum, B27, L-glutamine, and penicillin-streptomycin antibiotics. The neuronal cell cultures were kept in a humidified incubator (37 °C with 5% (vol/vol) CO_2_) for 7 days in vitro (DIV). After 3 days, the medium was changed to medium without FBS but supplemented with L-glutamine, B27, and penicillin-streptomycin antibiotics. The number of astrocytes did not exceed 10%, as determined by the content of the intermediate filament protein GFAP (glial fibrillary acidic protein). The experiments were conducted in compliance with European Union Legislation (Directive 2010/63/EU, amended by Regulation (EU) 2019/1010).

#### 2.1.2. Experimental Models

##### Hypoxia

To induce hypoxic conditions, the cell medium was replaced with a standard medium and placed in a prewarmed and humidified hypoxia modular incubator chamber (Billups-Rothenberg, Inc., San Diego, CA, USA) with 95% N_2_/5% CO_2_ for 6 h. The O_2_ level was measured with an oxygen analyzer (Greisinger, Germany) and reached less than 0.5%. After 6 h of hypoxic conditions, i.e., at the reoxygenation period, the culture medium was replaced immediately with standard medium for 18 h.

##### Ischemia

To induce ischemic conditions, the cell medium was replaced with medium without glucose and placed in a prewarmed and humidified hypoxia modular incubator chamber (Billups-Rothenberg, Inc., San Diego, CA, USA) with 95% N_2_/5% CO_2_ for 6 h. The O_2_ level was measured with an oxygen analyzer (Greisinger, Germany) and reached less than 0.5%. After 6 h of ischemic conditions, i.e., at the reoxygenation period, the culture medium was replaced immediately with standard medium for 18 h.

#### 2.1.3. Treatment

##### Co-Treatment

**Scheme 1:**
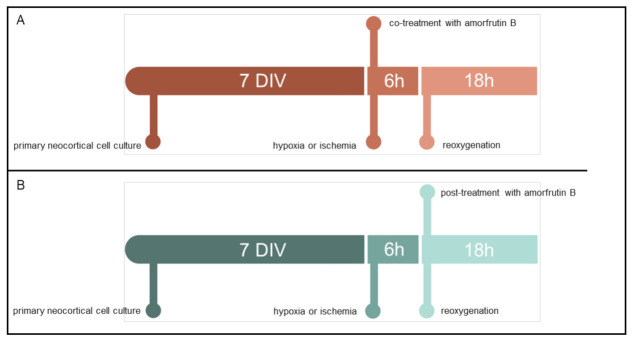
Experimental model of hypoxia and ischemia at 7 DIV in primary neocortical cell culture—co-treatment (**A**) and post-treatment (**B**).

The co-treatment experiments occurred when the cell cultures were treated simultaneously with hypoxic and ischemic episodes for 6 h, and the culture medium was changed to standard medium for 18 h reoxygenation (Scheme 1A). Cell cultures were treated with amorfrutin B at a concentration of 0.1–10 μM. During reoxygenation, cells were cultured in a humidified incubator (New Brunswick Scientific, Edison, NJ, USA).

##### Post-Treatment

After 6 h of hypoxic/ischemic conditions, i.e., at the reoxygenation period, the culture medium was replaced immediately with standard medium, and the treatment occurred for the next 18 h (Scheme 1B). Cell cultures were treated with amorfrutin B at a concentration of 0.1–10 μM. During reoxygenation, cells were cultured in a humidified incubator (New Brunswick Scientific, Edison, NJ, USA).

As the post-treatment paradigm reflects much better clinical and therapeutic aspects than the co-treatment paradigm, we chose the post-treatment paradigm for majority of experiments.

#### 2.1.4. Measurement of LDH Activity

To quantify hypoxia- and ischemia-induced cytotoxicity, the extent of LDH release was measured using a Cytotoxicity Detection Kit (Roche, Basil, Switzerland) as previously described [17]. After the experiment, cell-free supernatants were collected from each well and incubated with the appropriate reagent mixture for 30 min at room temperature, according to the manufacturer’s protocol. The intensity of red color was measured at a wavelength of 490 nm using an Infinite M200pro microplate reader (Tecan, Männedorf, Switzerland) and was proportional to the amount of LDH in the culture, which was, in turn, directly proportional to the number of dead or damaged cells. The data were analyzed with Tecan i-control software, normalized to the color intensity from vehicle-treated cells, and expressed as a percentage of the control value ± SEM. The absorbance of the blanks, which were no-enzyme controls, was subtracted from each value.

#### 2.1.5. Assessment of Cell Metabolic Activity

The viability of neurons after hypoxic and ischemic conditions was determined using the colorimetric MTT assay. Mitochondrial function was assessed through MTT reduction by oxidoreductase enzymes to purple formazan. MTT solution was added to the medium, and cell cultures were incubated at 37 °C. After 1 h, the medium was replaced with 100% DMSO to dissolve formazan crystals, and the intensity of the purple color was proportional to the number of viable cells. The absorbance was measured at 570 nm using an Infinite M200pro microplate reader, and the data were analyzed with i-control software. The results were normalized to the color intensity from vehicle-treated cells and are presented as a percentage of the control ± SEM.

#### 2.1.6. Measurement of the Degenerating Neurons

For the analysis of cytotoxicity in neuronal cell cultures after hypoxia and ischemia, Fluoro-Jade C (FJ-C) labeling was used, which is useful to label degenerating neurons using fluorochromatic dye. The stock solution was prepared as previously described [18] by mixing FJ-C with distilled water. Then, the culture medium was replaced with a prepared solution. After 1 h, the fluorescence of the FJ-C-labeled cells was measured using an excitation wavelength of 490 nm and an emission wavelength of 525 nm. The measurements were made using an Infinite M200PRO microplate reader, and the results were analyzed by i-control software. Data were normalized to the blank and presented as a percentage of the control ± SEM.

#### 2.1.7. Measurement of ROS Formation

The levels of hydrogen peroxide (H_2_O_2_) in neocortical neurons were measured after the experiment with the bioluminescent ROS-Glo™ H_2_O_2_ Assay (Promega, Madison, WI, USA). In general, the substrate reacts directly with H_2_O_2_ to generate a luciferin precursor. Adding Substrate Detection Solution causes the conversion to luciferin and provides the Ultra-Glo™ Luciferase to produce a light signal proportional to the amount of H_2_O_2_ in cultured cells. The bioluminescence was measured with a GloMax^®^ Navigator Microplate Luminometer (Promega, Madison, WI, USA), and the data were normalized to the bioluminescent signal intensity in the vehicle-treated cells and presented as a percentage of the control ± SEM.

#### 2.1.8. Estimation of DNA/RNA Oxidative Damage

The DNA/RNA Oxidative Damage (High Sensitivity) ELISA Kit (Cayman Chemical, Ann Arbor, MI, USA) was used to detect 8-OHdG in the samples after the experiment. This immunoassay consisted of binding the antibody-oxidatively damaged guanine complex to the goat polyclonal anti-mouse IgG. After adding Ellman’s reagent, there was an enzymatic reaction that resulted in a product with a distinct yellow color. The absorbance of this product was determined spectrophotometrically at 412 nm, and the intensity of color was inversely proportional to the amount of free 8-OHdG. The data were normalized to the vehicle-treated cells, and the results are presented as the 8-hydroxy-2′-deoxyguanosine concentration ± SEM.

#### 2.1.9. PPARγ Antagonist

To verify the involvement of PPARγ signaling in the neuroprotective action of amorfrutin B, the cultures were treated with a PPARγ antagonist (GW9662, 1 μM). To avoid nonspecific reactions, amorfrutin B and GW9662 were used at concentrations that did not affect LDH release under normoxic conditions. Amorfrutin B and PPARγ antagonist were originally dissolved in dimethyl sulfoxide (DMSO) and then diluted in a culture medium, resulting in DMSO concentrations below 0.1%.

During experiments with the use of PPARγ receptor antagonist GW9662, we decided to use both effective concentrations, i.e., 1 and 5 µM of amorfrutin B. This was because of uncertainty whether antagonist activity would dysregulate chosen parameter, i.e., LDH release in nonspecific way.

#### 2.1.10. Silencing of *Pparg* Using Small Interfering RNA (siRNA)

To inhibit *Pparg* expression in neuronal cell cultures, specific *Pparg* siRNA (50 nM) was applied (Santa Cruz Biotechnology Inc., Santa Cruz, CA USA). siRNA was added to medium without antibiotics containing the siRNA transfection reagent INTERFERin. After 7 h of transfection, the culture medium was replaced with a standard medium, and the cells were incubated until the next day of the experiment. Then, the cells were exposed to hypoxia or ischemia for 6 h and next treated with amorfrutin B during the reoxygenation. The negative siRNA did not lead to the specific degradation of any known cellular mRNA and was used as a control.

Gene silencing may affect cell survival nonspecifically. As both the 1 and 5 µM concentrations of amorfrutin B caused neuroprotection, though 5 µM amorfrutin B was more effective, we used both these concentrations of amorfrutin B to additionally confirm the specific actions of gene silencing.

#### 2.1.11. qPCR Analysis of mRNAs Specific to Genes Encoding *Hif1a*, *Pparg*, *Pgc1a*, and *Adipoq*

Total RNA was extracted from neocortical cells at 18 h post-treatment after hypoxia and ischemia with the RNeasy Mini Kit (Qiagen, Hilden, Germany) using the spin column-based method. The quantity of RNA was measured spectrophotometrically at 260 nm and 260/280 nm using a NanoDrop ND-1000 UV-Vis Spectrophotometer (Thermo Fisher Scientific, Waltham, MA, USA), and the RNA purity was determined when the A260/A280 ratio was approximately 2.0. Then, there was two-step qPCR involving reverse transcription and qPCR, which were run on a CFX 96 Real-Time PCR Detection System (Bio-Rad, Hercules, CA, USA). Total RNA was reverse transcribed with a High-Capacity cDNA Reverse Transcription Kit (Thermo Fisher Scientific, Waltham, MA, USA) in accordance with the manufacturers’ protocol. The collected cDNA was used as the PCR template and amplified with the TaqMan Gene Expression Master Mix kit (Thermo Fisher Scientific, Waltham, MA, USA) using TaqMan probes as primers for the specific genes encoding *Hif1a*, *Pparg*, *Pgc1a*, and *Adipoq*. The PCR process consisted of series of temperature changes: 2 min at 50 °C and 10 min at 95 °C, followed by 40 cycles of 15 s at 95 °C and 1 min at 60 °C. During the exponential phase, the threshold cycle (Ct) for each sample was set, and the delta delta Ct method was used for data analysis. The *Hprt* was selected as a reference gene using the following algorithms: geNorm, NormFinder, BestKeeper and delta Ct.

#### 2.1.12. Enzyme-Linked Immunosorbent Assays for PPARγ, PGC1α and ADIPOQ

Enzyme-linked immunosorbent assays (ELISAs) and the quantitative sandwich enzyme immunoassay technique were used to quantify the PPARγ, PGC1α, and ADIPOQ protein expression levels in the collected cell lysates after the experiment. The protein concentration was assessed using Bradford reagent (Bio-Rad Protein Assay) and bovine serum albumin as a standard. Each well of the 96-well plate was precoated with mouse antibodies specific for PPARγ, PGC1α, or ADIPOQ. The standards and samples were added to the wells with biotin-conjugated polyclonal antibodies specific for PPARγ, PGC1α, and ADIPOQ. Then, streptavidin-HRP (horseradish peroxidase) was added to the wells bound to the biotinylated antibodies, and during the washing step, the unbound streptavidin-HRP was washed away. The substrate solution caused a color change in proportion to the amount of proteins, and the addition of acidic stop solution terminated the reaction. The absorbance was measured at 450 nm using an Infinite M200PRO microplate reader, and the data were normalized to the color intensity from vehicle-treated cells and expressed as a percentage of the control value ± SEM.

#### 2.1.13. Western Blot Analysis

After 18 h of reoxygenation, the cells were lysed in ice-cold RIPA lysis buffer with a protease inhibitor cocktail. The lysates were sonicated and centrifuged at 15,000× *g* for 20 min at 4 °C. The supernatants were collected, and the protein concentration was assessed using Bradford reagent (Bio-Rad Protein Assay) and bovine serum albumin as a standard. The cell lysates containing 30 μg of the total protein were reduced and denatured by boiling each sample in a 2× Laemmli sample buffer at 95 °C for 5 min. The molecular weight marker and samples were loaded into the wells of a 10% SDS-polyacrylamide gel, and the proteins were electrophoretically separated using a Bio-Rad Mini-Protean II Electrophoresis Cell. After separation, the proteins were electrotransferred from the gel to polyvinylidene fluoride (PVDF) membranes (Millipore, Burlington, MA, USA) using the Bio-Rad Mini Trans-Blot apparatus. To prevent nonspecific antibody binding, the membranes were washed and blocked with 5% dried milk and 0.2% Tween-20 in 0.02 M Tris-buffered saline (TBS) for 2 h. The membranes were incubated overnight (at 4 °C) with one of the following appropriate dilutions of primary antibodies: anti-HIF1α rabbit polyclonal antibody (diluted 1:100), anti-PPARγ rabbit polyclonal antibody (diluted 1:100) or anti-β-actin mouse monoclonal antibody (diluted 1:3000) in TBS/Tween. After incubation with primary antibodies, the membranes were washed with TBS and 0.02% Tween 20 and incubated for 2 h with HRP-conjugated goat anti-rabbit IgG or goat anti-mouse IgG secondary antibodies (Santa Cruz Biotechnology, Santa Cruz, CA, USA) diluted at 1:1000 in TBS/Tween. To control the amount of denatured protein loaded onto the gel, the membranes were stripped and reprobed with an anti-β-actin antibody. The signals were developed by enhanced chemiluminescence (ECL) using BM Chemiluminescence Blotting Substrate (Roche Diagnostics, Basil, Switzerland) and visualized using a Luminescent Image Analyzer Fuji-Las 4000 (Fuji, Japan). Immunoreactive bands were quantified using a MultiGauge V3.0 image analyzer and normalized to β-actin. The data are presented as a percentage of the control value ± SEM, and pictures of exemplary stripes are shown.

#### 2.1.14. Immunofluorescence Staining of PPARγ and MAP2

To visualize the cellular localization of PPARγ and confirm the neuronal nature of the neocortical cells, immunofluorescence labeling, and confocal microscopy were used. The neocortical cells were cultured on glass coverslips and subjected to immunofluorescence double labeling at 7 DIV. Cultured cells were fixed for 15 min at room temperature with a 4% paraformaldehyde solution in PBS, and then cells were incubated for 1 h in a blocking buffer containing 5% normal donkey serum and 0.3% Triton X-100 in 0.01 M PBS. After that, the neurons were incubated for 24 h at 4 °C with primary antibodies: anti-PPARγ mouse monoclonal (diluted 1:50) and anti-MAP2 rabbit polyclonal (diluted 1:100) antibodies (Santa Cruz Biotechnology, Santa Cruz, CA, USA). This step was followed by an overnight incubation in a mixture of the following secondary antibodies (Thermo Fisher Scientific, Waltham, MA, USA): Alexa Fluor Plus 488-conjugated anti-mouse IgG (1:300) and Alexa Fluor Plus 647-conjugated anti-rabbit IgG (1:300). The microscopic preparations were then washed with PBS, mounted, and cover-slipped. For viewing slides, a Leica TCS SP8 WLL confocal laser scanning microscope (DMi8-CS, Leica Microsystem, Wetzlar, Germany) was used.

To quantify the immunofluorescence signal corresponding to PPARγ expression level, the frequency of the brightest pixels in the region of interest (ROI) has been measured. To assess pixel intensity the ImageJ software has been used.

#### 2.1.15. Analyses of DNA Methylation

##### Global DNA Methylation

The DNA from the cell cultures was extracted with the use of Quick-gDNA™ MicroPrep Kit (Zymo Research, Irivine, CA, USA) and quantified spectrophotometrically using the NanoDrop ND-1000 UV-Vis Spectrophotometer (Thermo Fisher Scientific, Waltham, MA, USA). The methylation pattern was evaluated using an Imprint Methylated DNA Quantification Kit (Sigma-Aldrich, Saint Louis, MO, USA). According to the manufacturer’s instructions, 50 ng of purified DNA was added to each well where the methylated DNA was detected by capture and detection antibodies. The reaction for color change was monitored using the developing solution. The absorbance of the colorimetric reaction product was measured at 450 nm using an Infinite M200PRO microplate reader.

##### The *Pparg* Gene Specific Methylation

Genomic DNA was extracted using a Quick-gDNA™ MicroPrep Kit (Zymo Research, Irvine, CA, USA), and the quantity of DNA was determined spectrophotometrically at 260 nm and 260/280 nm with the use of a NanoDrop ND-1000 UV-Vis Spectrophotometer (Thermo Fisher Scientific, Waltham, MA, USA). Then, denaturation and complete bisulfite conversion of GC-rich DNA were accomplished by an EZ DNA Methylation-Gold™ Kit (Zymo Research, Irvine, CA, USA). The samples were eluted in a 10 μL volume and subjected to qPCR (MethyLight) using the EpiTect MethyLight PCR Kit (Qiagen, Hilden, Germany). The methylation regions in *Pparg* gene were verified in a CpG hot spots in the 5’ flanking sequence (2000 bp). The Methyl Primer Express Software 1.0. has been used to design methylated and unmethylated target sequences and to design the primers. Fully methylated and fully unmethylated TaqMan probes were designed for *Pparg* promoters, and the internal reference set for the *Hprt* gene was designed to control the input DNA. The unmethylation-specific TaqMan probe was bound to VIC^®^, while the methylation-specific TaqMan probe was linked to FAM™ as a 5′ reporter dye. The release of these indicators and determination of the ratio of measured Ct values with both fluorescence dyes enable the assessment of methylation status. To calculate the methylation degree in samples, the Ct determined with each of both dyes were used: percentage of methylation [%]: C_meth_ = 100/[1 + 2(ΔCt_meth_ − ΔCt_unmeth_)].

#### 2.1.16. Estimation of Histone Deacetylase and Acetyltransferase Activities

##### Histone Deacetylase (HDAC) Activity

To verify HDAC activity, the Histone Deacetylase Assay Kit (Sigma-Aldrich, Saint Louis, MO, USA) was applied. The assay kit is based on a two-step enzymatic reaction: I. deacetylation of the acetylated lysine side chain by the HDAC-containing sample and II. cleavage of the deacetylated substrate by the developer solution. The fluorescence was measured with an Infinite M200PRO microplate reader at λex = 365 nm/λem = 460 nm, and the release of the free highly fluorescent group was proportional to the deacetylation activity. The kit included positive (HeLa cell lysate) and negative (trichostatin A) controls.

##### Sirtuins Activity

The Sirtuin Activity Assay Kit (Sigma-Aldrich, Saint Louis, MO, USA) was used to estimate the sirtuins activity in the samples. Sirtuins in the presence of NAD+ are able to form deacetylated p53-AFC substrates, nicotinamide, and O-acetyl-ADP ribose. According to the protocol, adding ‘developer solution’ caused the cleavage of the deacetylated p53-AFC substrate and the release of the fluorescent group, which was fluorometrically detected at λex = 400 nm/λem = 505 nm with an Infinite M200PRO microplate reader. The p53-AFC substrate can also be deacetylated by nonsirtuin HDACs; therefore, trichostatin A was added to the solution to inhibit nonsirtuins activities in the samples. The positive control has also been included.

#### 2.1.17. Estimation of Histone Acetyltransferase (HAT) Activity

HAT activity was measured using the HAT Activity Fluorometric Assay Kit (Sigma-Aldrich, Saint Louis, MO, USA). According to the manufacturers’ protocol, the reaction product of histone acetyltransferase activity reacts with the developer solution, which results in the fluorescent product of the reaction measured using an Infinite M200PRO microplate reader (λex = 535/λem = 587 nm). The obtained values were proportional to the HAT activity in the samples. The assay contained HeLa nuclear extract, which was used as a positive control.

### 2.2. Data Analysis

The statistical analysis was performed based on raw data expressed as the absorbance or fluorescence units per well containing 50,000 cells for the LDH, MTT, FJ-C, ROS formation, DNA/RNA oxidative damage assays; the fluorescence units per 1.5 million cells for qPCR, global DNA methylation, HAT, HDAC, and sirtuin activity; the mean optical density per 30 μg of protein for Western blot assays; the pg of PPARγ, PGC1α, and ADIPOQ per μg of total protein for the ELISAs. To determine overall significance, an analysis of variance (ANOVA) was used, previously preceded by Levene’s test of homogeneity of variances. The differences between the control (vehicle-treated cells) and experimental groups were defined with a post hoc Newman-Keuls test. Significant differences were marked in the following ways: * *p* < 0.05, **p < 0.01, and *** *p* < 0.001 (compared to the control groups), # *p* < 0.05, ## *p* < 0.01, and ### *p* < 0.001 (compared to the cultures exposed to hypoxia), ^ *p* < 0.05, ^^ *p* < 0.01, and ^^^ *p* < 0.001 (compared to the cultures exposed to ischemia). The results for DNA/RNA oxidative damage, qPCR, ELISAs, DNA methylation, and histone deacetylase activity are expressed as the mean ± SEM of three independent experiments. The results for LDH release, MTT, FJ-C, ROS activity, Western blot analysis, and HAT activity are presented as the percentage of the control ± SEM of three independent experiments. The number of replicates ranged from 6 to 12.

Under normoxic conditions, amorfrutin B did not induce changes in: MTT, Fluoro-Jade C, ROS activity, 8-OHdG level, protein and mRNA expression levels (except for slight increase in *Pgc1a* mRNA level), epigenetic status of neuronal cells, and immunofluorescence intensity (Supplementary Materials—Table S1). Hypoxic or ischemic conditions and exposure to amorfrutin B (1 and 5 μM) post-treatment did not contribute to changes in caspase-3 activity (Figure S1)

## 3. Results

### 3.1. The Effects of Amorfrutin B on Hypoxia- and Ischemia-Induced Lactate Dehydrogenase (LDH) Release in Neocortical Cell Cultures

**Figure 1:**
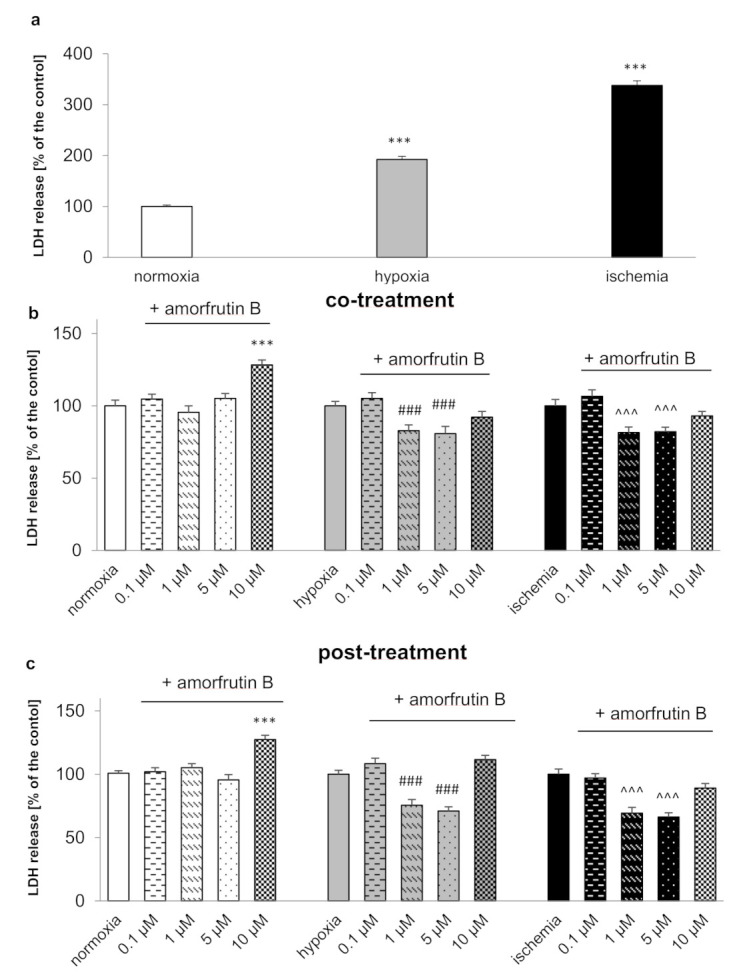
Time course and concentration-dependent response to hypoxia or ischemia and amorfrutin B treatment in mouse neocortical cells at 7 DIV. In both the co-treatment (treatment simultaneously with 6 h of hypoxia or ischemia) and post-treatment (treatment after 6 h of hypoxia or ischemia) paradigms, amorfrutin B (1 and 5 μM) decreased hypoxia- and ischemia-induced LDH release, while under normoxic conditions, amorfrutin B (0.1–5 μM) did not affect this parameter (**a**–**c**). The results are presented as a percentage of the control ± SEM of 3 independent experiments, consisting of 8–12 replicates per group. *** *p*  <  0.001 compared to the control group, ### *p* < 0.001 compared to the cultures exposed to hypoxia, ^^^ *p* < 0.001 compared to the cultures exposed to ischemia.

In this study, a model of 6 h hypoxia or ischemia followed by 18 h of reoxygenation was applied. Hypoxia and ischemia conditions induced LDH release to 192% and 338% of the normoxic value, respectively (Figure 1a).

In the paradigm of co-treatment, i.e., when the amorfrutin B was added at the same time as the injury started, the effects were as follows: (i) in the hypoxic model, amorfrutin B (1 µM and 5 µM) co-treatment inhibited LDH release to 83% (17% decrease) and 81% (19% decrease) of the hypoxia value, respectively; (ii) in the ischemic model, amorfrutin B (1 µM and 5 μM) co-treatment also reduced ischemia-induced LDH activity to 82% (18% decrease), Figure 1b.

In the paradigm of post-treatment, i.e., when amorfrutin B was added during the reoxygenation period, the following effects were observed: (i) in the hypoxic model, amorfrutin B (1 µM and 5 µM) post-treatment inhibited LDH release to 75% (25% decrease) and 71% (29% decrease) of the hypoxia value, respectively; (ii) in the ischemic model, amorfrutin B (1 µM and 5 μM) post-treatment also reduced ischemia-induced LDH activity to 69 and 66% (31 and 34% decrease), respectively (Figure 1c).

Under normoxic conditions, amorfrutin B used at concentrations ranging from 0.1 to 5 μM did not change LDH release, but amorfrutin B at a concentration of 10 μM was cytotoxic to neocortical cells. Therefore, for further experiments, we did not use 10 μM amorfrutin B.

We chose the post-treatment paradigm for majority of experiments because it reflects much better clinical aspects than co-treatment paradigm. As the most promising post-treatment paradigm was with the 5 µM concentration (Figure 1c), it has been used in the next experiments.

### 3.2. The Effects of Amorfrutin B on the Viability and the Degeneration of the Neuronal Cells

#### 3.2.1. The Impact of Amorfrutin B on the Viability of Neuronal Cells under Hypoxic and Ischemic Conditions

**Figure 2:**
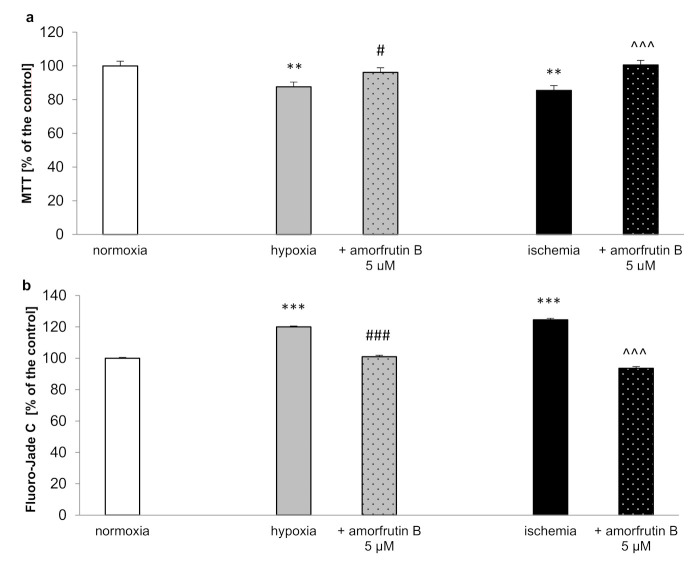
Amorfrutin B (5 μM) reversed the hypoxic- and ischemic-induced loss in viability and reduced neurodegeneration of primary neocortical cell cultures. Hypoxia and ischemia decreased the viability (**a**) and increased the degeneration (**b**) of neuronal cells, and amorfrutin B manifested its neuroprotection by elevated levels of viable cells and reduced neurodegeneration, respectively. The results are presented as a percentage of the control ± SEM of 3 independent experiments, consisting of 8–12 replicates per group. ** *p*  <  0.01 and *** *p*  <  0.001 compared to the control group, # *p* < 0.05 and ### *p* < 0.001 compared to the cultures exposed to hypoxia, ^^^ *p* < 0.001 compared to the cultures exposed to ischemia.

Six hours of hypoxic or ischemic conditions reduced the viability of neuronal cells from the control value (100%) to 88% and 85%, respectively, as measured using MTT (3-(4,5-dimethylthiazol-2-yl)-2,5-diphenyltetrazolium bromide). Exposure to 5 μM amorfrutin B for 18 h of reoxygenation after hypoxia and ischemia resulted in an improvement in mitochondrial activity and an increased number of viable cells. Amorfrutin B administration increased cell survival after hypoxia and ischemia to 97% (9% increase) and 100% (15% increase) of the control value, respectively (Figure 2a).

#### 3.2.2. Amorfrutin B Reduced the Degeneration of Neuronal Cells Caused by Hypoxia and Ischemia

Fluoro-Jade C (FJ-C) labeling showed that cell degeneration levels due to hypoxia and ischemia reached 119% and 124% of the control value, respectively. In neocortical cells, amorfrutin B (5 μM) used in the post-treatment paradigm reduced the extent of neurodegeneration in both the hypoxic and ischemic models. After amorfrutin B application, the FJ-C indicator decreased 18% compared to hypoxia (101% of the control value) and 30% compared to ischemia (94% of the control value), Figure 2b.

### 3.3. The Effects of Amorfrutin B on the Oxidative Stress

#### 3.3.1. The Effects of Amorfrutin B on ROS Formation under Hypoxic and Ischemic Conditions

**Figure 3:**
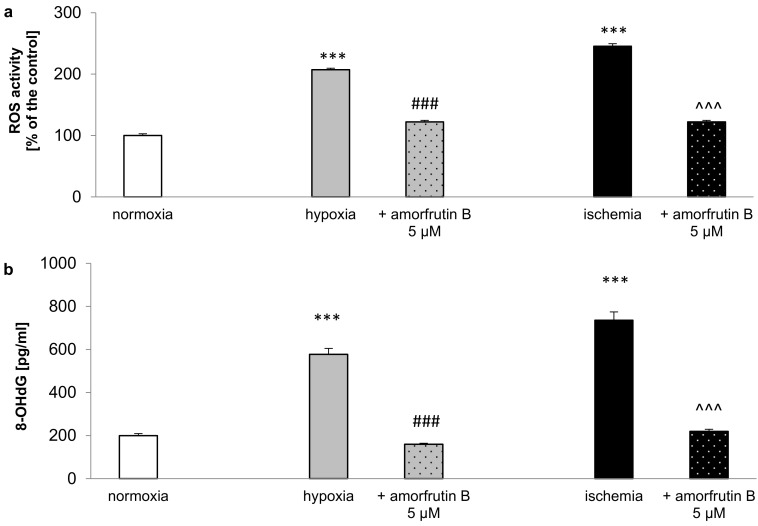
Amorfrutin B (5 μM) post-treatment reduced the oxidative stress resulting from hypoxia and ischemia in neuronal cells. There was an increase in reactive oxygen species formation and 8-OHdG in the hypoxic and ischemic models, and amorfrutin B diminished the activity of these markers in both cases (**a**,**b**). The ROS activity results are presented as a percentage of the control ± SEM, and the 8-OHdG level results are presented as the mean ± SEM. There were 3 independent experiments, consisting of 10–12 replicates per group. *** *p*  <  0.001 compared to the control group, ### *p* < 0.001 compared to the cultures exposed to hypoxia, ^^^ *p* < 0.001 compared to the cultures exposed to ischemia.

In both the hypoxic and ischemic models, there were statistically significant increases in ROS activity. ROS formation reached 207% and 245% of the control value during hypoxia or ischemia, respectively. Amorfrutin B (5 μM) reduced oxidative stress markers in both models; there was a reduction in ROS activity to 122% of the control value (Figure 3a).

#### 3.3.2. Hypoxia and Ischemia Caused DNA/RNA Oxidative Damage, Which Was Reduced by Amorfrutin B

DNA/RNA oxidative damage was assessed by measuring 8-hydroxy-2′ deoxyguanosine (8-OHdG) levels in two models: after 6 h of hypoxia/18 h of reoxygenation and 6 h of ischemia/18 h of reoxygenation in neuronal cell cultures (Figure 3b). In the control group, there was 200 pg/mL (100%) 8-OHdG. After hypoxia, post-treatment with amorfrutin B (5 μM) reduced the 8-OHdG level from 577 pg/mL (289% of the control value) to 159 pg/mL (72% decrease). Moreover, after ischemia, amorfrutin B (5 µM) caused a reduction in the 8-OHdG level from 736 pg/mL (368% of the control value) to 219 pg/mL (70% decrease).

### 3.4. Influence of PPARγ Antagonist on the Effect of Amorfrutin B in Neuronal Cells Exposed to Hypoxia and Ischemia

**Figure 4:**
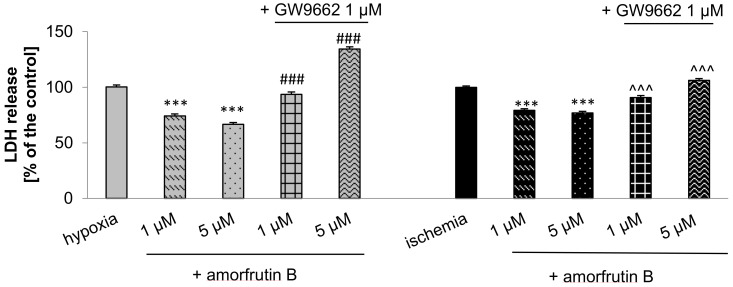
PPARγ antagonist (GW9662) reduced the neuroprotective effects of 1 and 5 μM amorfrutin B in the models of hypoxia and ischemia, which was manifested by elevated levels of LDH release. Neuronal cells were treated with GW9662 (1 μM) 6 h after hypoxia or ischemia for 40 min and subjected to amorfrutin B (1 or 5 μM) for the next 18 h of reoxygenation. The results are presented as a percentage of the control ± SEM of 3 independent experiments, consisting of 6–8 replicates per group. *** *p*  <  0.001 compared to the control group, ### *p*  < 0.001 compared to the cultures exposed to hypoxia, ^^^ *p* < 0.001 compared to the cultures exposed to ischemia.

A PPARγ antagonist (GW9662) was used at a 1 μM concentration that did not alter LDH release under normoxic conditions. In this study, GW9662 reduced the neuroprotective effects of 1 and 5 μM amorfrutin B in the model of hypoxia, which was manifested by elevated levels of LDH release to 94% and 134% of the control value, respectively, i.e., 20% and 100% increases. Under ischemic conditions, amorfrutin B reduced LDH release to 79% (1 μM) and 76% (5 μM) of the normoxic value. GW9662 reversed these protective effects by enhancing LDH release up to 91–106% of the control value (Figure 4).

### 3.5. Effect of Amorfrutin B on Hypoxia- and Ischemia-Induced LDH Release in Pparg siRNA-Transfected Neocortical Cells

**Figure 5:**
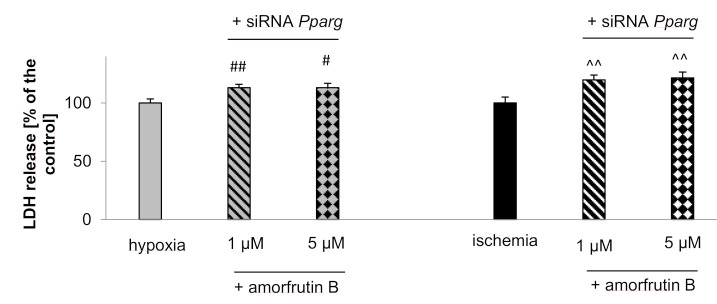
In *Pparg* siRNA-transfected cells, amorfrutin B (1 or 5 μM) lost the neuroprotective potential that was manifested by increased LDH release levels in both hypoxic and ischemic models. Neuronal cell cultures were transfected with 50 nM *Pparg* siRNA in INTERFERin-containing medium without antibiotics for 7 h. Then, the cells were exposed to hypoxia or ischemia for 6 h and next treated with amorfrutin B during the reoxygenation. The results were normalized to the absorbance in negative control siRNAs. The results are presented as a percentage of the control ± SEM of 3 independent experiments, consisting of 5–8 replicates per group. # *p* < 0.05 and ## *p* < 0.01 compared to the cultures exposed to hypoxia, ^^ *p* < 0.01 compared to the cultures exposed to ischemia.

In *Pparg* siRNA-transfected cells, amorfrutin B lost its neuroprotective potential. After transfection and treatment with amorfrutin B (1 or 5 μM), LDH reached 113% of the control value under hypoxic conditions. Moreover, in *Pparg* siRNA-transfected cells in the ischemic model, LDH activity reached 119%-121% (both concentrations of amorfrutin B) of the control value (Figure 5). The effectiveness of mRNA silencing was verified through the measurement of *Pparg* mRNAs using qPCR. Based on our previous study, after the silencing of *Pparg* with the specific siRNAs, the *Pparg* mRNA concentration has been reduced by 31% compared to the non-transfected wild-type cells [19].

### 3.6. Effects of Amorfrutin B on the mRNA Expression Levels of Hif1a, Pparg, Pgc1a, and Adipoq in Models of Hypoxia and Ischemia

The mRNA expression levels were measured with quantitative polymerase chain reaction (qPCR). In our study, a 6 h exposure to hypoxia enhanced the hypoxia-inducible factor 1 alpha (*Hif1a*) mRNA expression level to 1.26-fold than under normoxic conditions. *Pparg* and PPARγ-dependent adiponectin (*Adipoq*) mRNA expression levels were also stimulated to 2.43-fold and 3.68-fold, respectively. Hypoxia did not change the expression level of peroxisome proliferator activated receptor gamma coactivator 1 alpha (*Pgc1a*) mRNA in neocortical cells. During hypoxia, amorfrutin B (5 μM) post-treatment decreased the expression levels of *Hif1a* mRNA to 0.97-fold (23% decrease) and *Pgc1a* mRNA to 0.72-fold (28% decrease). In turn, amorfrutin B (5 µM) increased the mRNA expression level of *Pparg* to 1.84-fold compared to normoxia, but it did not change the mRNA expression level of *Adipoq* mRNA.

**Figure 6:**
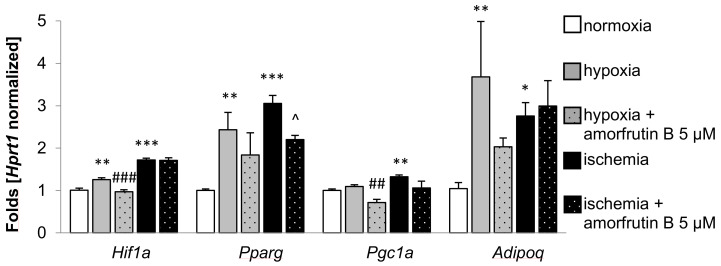
Alterations in the mRNA expression levels of *Hif1a*, *Pparg*, *Pgc1a* and *Adipoq* in response to hypoxia or ischemia and amorfrutin B (5 μM) treatment in neocortical cell cultures. The results are presented as a mean ± SEM. There were 3 independent experiments, consisting of 6 replicates per group. * *p* < 0.05, ** *p* < 0.01, and *** *p* < 0.001 compared to the control group, ## *p* < 0.01, ### *p* < 0.001 compared to the cultures exposed to hypoxia, ^ *p* < 0.05 compared to the cultures exposed to ischemia.

A 6 h exposure to ischemia enhanced the expression level of all studied genes, i.e., *Hif1a* mRNA to 1.72-fold, *Pparg* mRNA to 3.06-fold, *Pgc1a* mRNA to 1.32-fold, and *Adipoq* mRNA to 2.76-fold compared to the normoxia control group (1.00-fold). Under ischemic conditions, amorfrutin B (5 μM) post-treatment changed only the *Pparg* mRNA expression level to 2.20-fold, which was a decrease of 28% compared with the ischemic group. Amorfrutin B treatment did not significantly change the expression levels of *Pgc1a*, *Hif1a*, and *Adipoq* mRNA. All values were normalized to hypoxanthine-guanine phosphoribosyltransferase (*Hprt*), Figure 6.

### 3.7. Effects of Amorfrutin B on the Protein Expression Levels of HIF1α, PPARγ, PGC1α, and ADIPOQ in Models of Hypoxia and Ischemia

**Figure 7:**
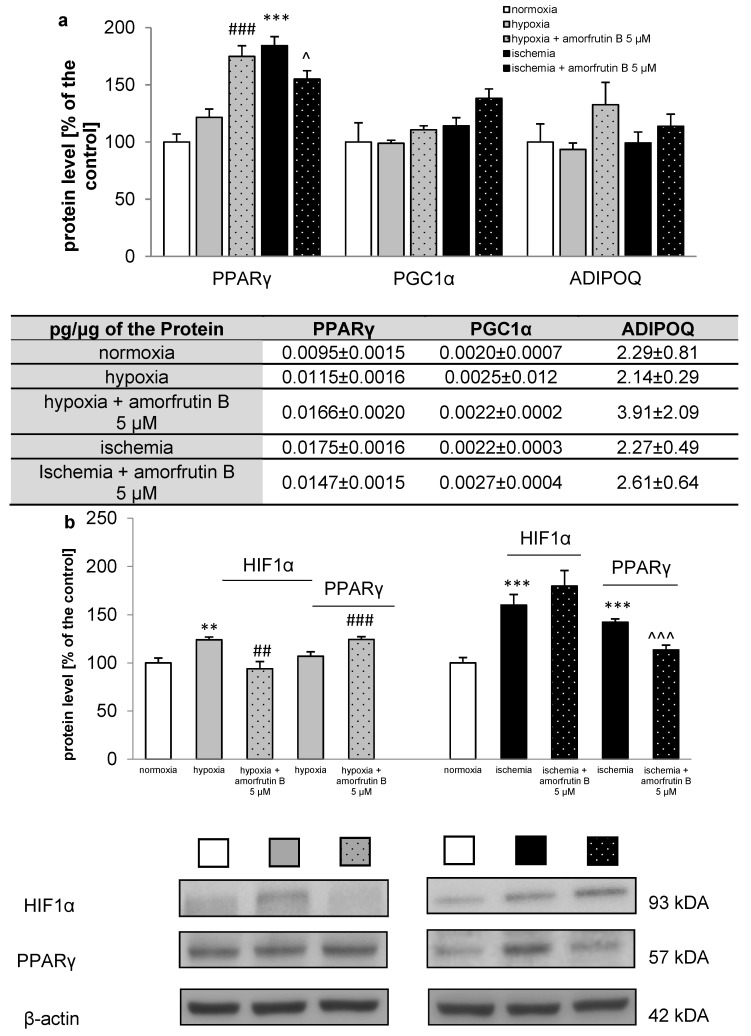
ELISA (**a**) and Western blot (**b**) showed that hypoxia or ischemia and amorfrutin B (5 μM) post-treatment changed the protein expression levels of HIF1α and PPARγ but did not affect the protein expression levels of PGC1α and ADIPOQ. The results are presented as a mean ± SEM. There were 3 independent experiments, consisting of 5–6 replicates per group. ** *p* < 0.01, and *** *p* < 0.001 compared to the control group, ## *p* < 0.01, ### *p* < 0.001 compared to the cultures exposed to hypoxia, ^ *p* < 0.05, ^^^ *p* < 0.001 compared to the cultures exposed to ischemia.

Enzyme-linked immunosorbent assays (ELISAs) showed that the protein levels of PPARγ, PGC1α, and ADIPOQ in control neuronal cells reached 0.0095, 0.0020, and 2.29 pg per µg of total protein, respectively. After hypoxia, the level of PPARγ reached 0.0115 pg, and amorfrutin B (5 μM) increased this value to 0.0166 pg per µg of total protein (44% increase). In response to ischemic conditions, the PPARγ protein expression level increased from 0.0095 pg to 0.0175 pg (84% increase), and exposure to amorfrutin B decreased the PPARγ level to 0.014 pg (20% decrease compared to ischemia). There were no statistically significant changes in PGC1α and ADIPOQ protein expression levels under hypoxic and ischemic conditions, and amorfrutin B post-treatment also did not affect the levels of these proteins (Figure 7a).

Western blot analysis showed that hypoxic conditions induced an increase in HIF1α protein expression levels from 100% to 124% of the normoxic value (24% increase) and amorfrutin B (5 μM) post-treatment decreased this value to 94% (25% decrease). In this study paradigm, hypoxia did not affect the protein expression of PPARγ; however, amorfrutin B treatment increased the PPARγ level to 124% of the normoxic value (24% increase). Under ischemic conditions, there was also an increase in HIF1α protein expression to 160% of the normoxic value (60% increase), but exposure to amorfrutin B did not significantly change this value. The same experimental conditions caused an increase in the PPARγ protein level to 142% (42% increase), and amorfrutin B reduced this level to 114% of the normoxic value (Figure 7b).

### 3.8. Confocal Microscopic Analysis of PPARγ and MAP2 Localization in Neuronal Cells

**Figure 8:**
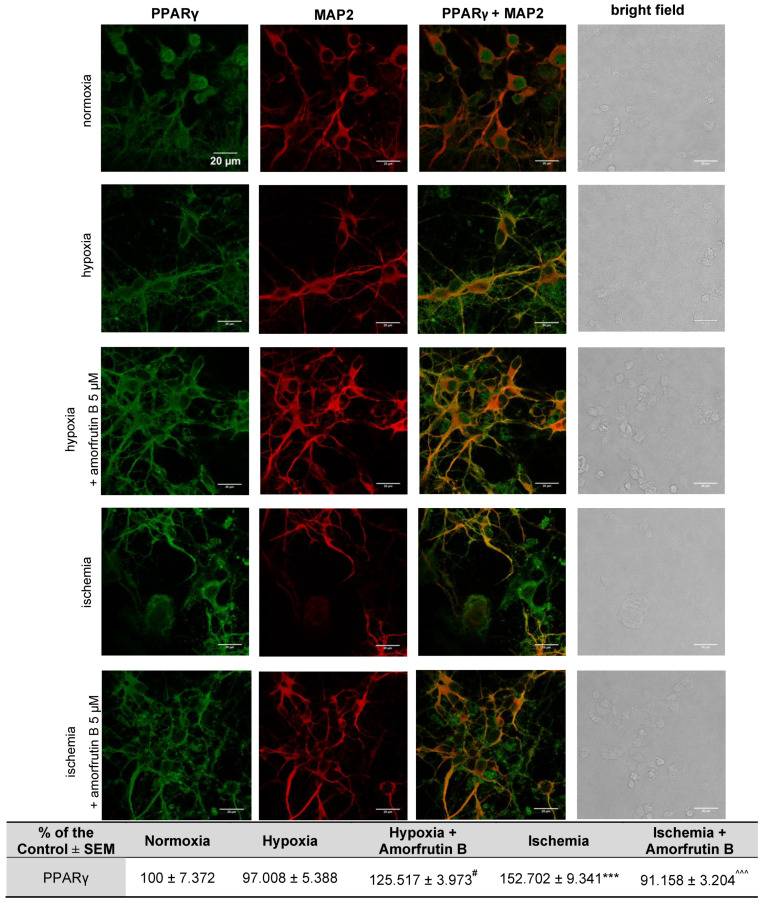
The effect of hypoxia or ischemia and amorfrutin B (5 μM) post-treatment on immunofluorescence staining of PPARγ (green staining) and MAP2 (red staining). The bright-field images were also shown. MAP2 confirmed the presence of neuronal cells and showed hypoxia- and ischemia-induced inhibition of neurite outgrowth, reduced by amorfrutin B administration. Staining also showed the distribution of PPARγ in neuronal cells. The table shows the average pixel intensity for region of interest (ROI). The results are presented as a percentage of the control ± SEM. *** *p* < 0.001 compared to the control group, # *p* < 0.05 compared to the cultures exposed to hypoxia, ^^^ *p* < 0.001 compared to the cultures exposed to ischemia. The number of replicates in each group was 5.

Immunofluorescence staining and confocal microscopy showed that PPARγ receptors (green staining) were localized in neuronal cells at 7 days in vitro (DIV). Exposure to hypoxic conditions did not change the PPARγ specific staining, but it was enhanced after amorfrutin B (5 μM) post-treatment. Moreover, PPARγ-specific labeling showed an increase in staining after ischemic conditions, which was reduced after exposure to amorfrutin B. Microtubule-associated protein 2 (MAP2) (red staining) indicated the location of neuronal cells and showed that in comparison to the control, there was hypoxia-induced inhibition of neurite outgrowth, which was even more advanced under ischemic conditions. In both cases, amorfrutin B significantly improved cell viability (Figure 8).

The expression levels of PPARγ have been quantified using basic intensity quantification method. According to these data, hypoxia did not affect PPARγ expression level (97% of the control) while post-treatment with amorfrutin B increased its expression to 125% of the control value. In the ischemic condition, PPARγ expression level enhanced by 53% (153% of the control value) and amorfrutin B restored its expression to over 90% of the control (Figure 8).

### 3.9. Impact of Amorfrutin B on DNA Methylation under Hypoxic and Ischemic Conditions

#### 3.9.1. Global DNA Methylation

**Figure 9:**
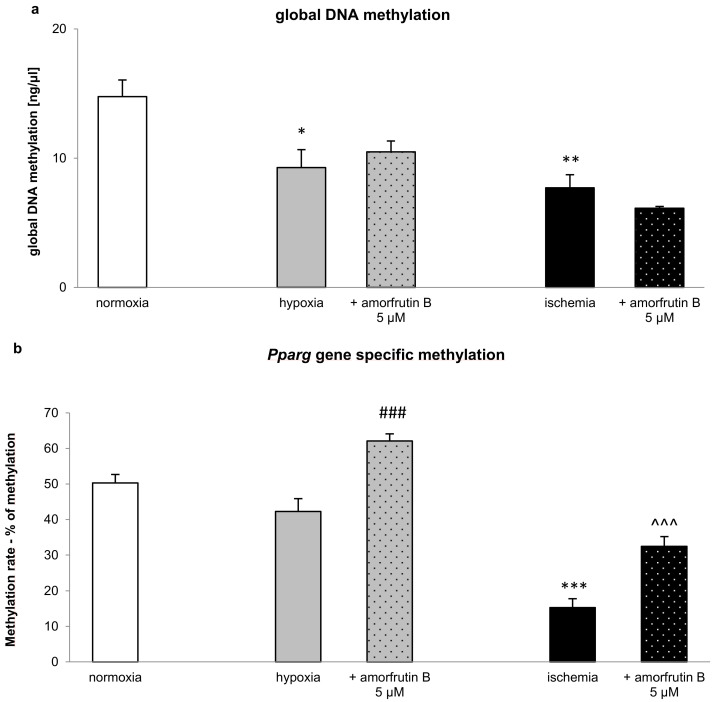
Hypomethylation of global DNA (**a**) and alterations in gene specific methylation of *Pparg* (**b**) after hypoxia or ischemia and exposure to amorfrutin B (5 μM). The results are presented as a mean ± SEM. There were 3 independent experiments, consisting of 6 replicates per group. * *p* < 0.05, ** *p* < 0.01, and *** *p* < 0.001 compared to the control group, ### *p* < 0.001 compared to the cultures exposed to hypoxia, ^^^ *p* < 0.001 compared to the cultures exposed to ischemia.

In control neuronal cells (normoxic conditions), the global DNA methylation value reached 15 ng/μL (100%). Hypoxia and ischemia caused hypomethylation, decreasing global DNA methylation to 9 ng/μL (40% decrease) and 8 ng/μL (47% decrease), respectively. Amorfrutin B post-treatment had no statistically significant influence on this effect as global DNA methylation reached a value of 10 ng/μL under hypoxic conditions and 6 ng under ischemic conditions (Figure 9a).

#### 3.9.2. The *Pparg* Gene Specific Methylation

In neuronal cell cultures, the methylation rate in the control group under normoxic conditions reached 50%. Our study showed that in response to hypoxia, there was no statistically significant change in *Pparg* methylation, but amorfrutin B (5 μM) post-treatment caused an increase in methylation from 42% (after hypoxia) to 62% (hypermethylation). In turn, *Pparg* methylation decreased from 50% in the control group to 15% (hypomethylation) under ischemic conditions, and after amorfrutin B treatment, there was an increase to 32% (hypermethylation), Figure 9b.

### 3.10. Histone Deacetylase and Histone Acetyltransferase Activities in a Model of Hypoxia or Ischemia and the Response to Amorfrutin B

#### 3.10.1. HDAC Activity

**Figure 10:**
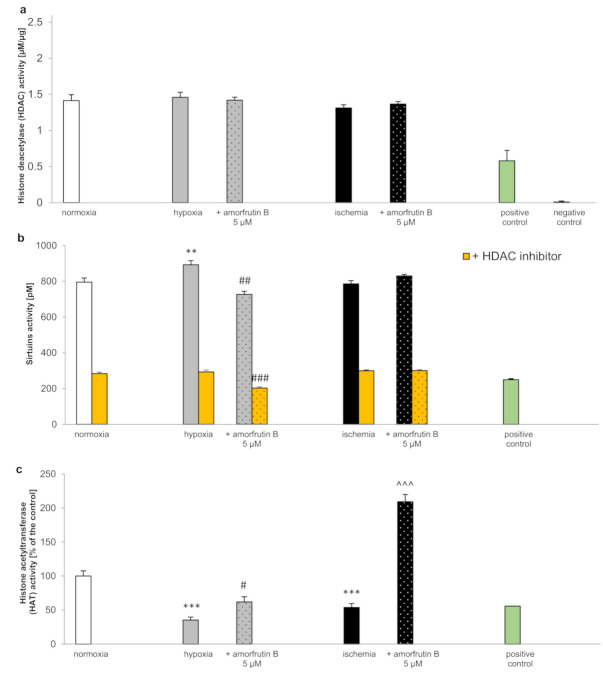
Hypoxic or ischemic conditions and exposure to amorfrutin B (5 μM) post-treatment did not contribute to changes in histone deacetylase (HDAC) activity (**a**) but altered sirtuins and nonsirtuins action (**b**) and histone acetyltransferase (HAT) activity (**c**). A positive control contained HeLa cell lysate for HDAC, an undefined sample for sirtuins, and a HeLa nuclear extract for HAT. Negative control had no cells, and an HDAC inhibitor (trichostatin A) was added to inhibit nonsirituin activities in the samples. The results are presented as the mean ± SEM (HDAC and sirtuins activity) or as a percentage of the control ± SEM (HAT activity). There were 3 independent experiments, consisting of 5–6 replicates per group. ** *p* < 0.01, and *** *p* < 0.001 compared to the control group, # *p* < 0.05, ## *p* < 0.01, ### *p* < 0.001 compared to the cultures exposed to hypoxia, ^^^ *p* < 0.001 compared to the cultures exposed to ischemia.

In this study, hypoxic and ischemic conditions did not significantly affect HDAC activity, reaching approximately 1.4 μM/μg. Additionally, in response to amorfrutin B exposure, there were no changes in this parameter in the studied models. Positive and negative controls were estimated at levels of 0.58 μM/μg (HeLa cell lysate) and 0.01 μM/μg (HDAC inhibitor-trichostatin A), respectively (Figure 10a).

#### 3.10.2. Sirtuins Activity

In response to hypoxia, sirtuins activity increased from a control value of 796 pM (100%) to 893 pM (12% increase), and amorfrutin B post-treatment decreased this value to 727 pM (19% decrease). Although hypoxic conditions did not affect nonsirituin HDAC activity (trichostatin A-treated cells), the addition of amorfrutin B decreased this parameter from 293 pM to 203 pM (31% decrease). In ischemia, treatment with amorfrutin B did not affect the activities of sirtuins and nonsirituin HDACs. A positive control was provided by the manufacturer and displayed a level of 250 pM (Figure 10b).

#### 3.10.3. HAT Activity

Hypoxia and ischemia decreased HAT activity, which was reduced to 35% and 54% of the control value, respectively. Amorfrutin B (5 µM) treatment increased the HAT activity to 62% under hypoxic conditions and 209% under ischemic conditions of the control value. In the HeLa nuclear extract used as a positive control, HAT activity was estimated at 56% (Figure 10c).

## 4. Discussion

There is an urgent need to establish a new pharmacotherapy that would have a wide window, i.e., wider than 4.5 h for rt-PA, to effectively treat hypoxia- and ischemia-induced brain damage. In this study, we demonstrated for the first time that amorfrutin B, a selective modulator of PPARγ, can protect mouse brain neurons from hypoxia- and ischemia-induced degeneration when applied at 18 h post-treatment, which started at a 6 h delay from hypoxia and ischemia. Furthermore, PPARγ antagonist and *Pparg* mRNA silencing with specific siRNA abolished the neuroprotective effect of amorfrutin B, which points to agonistic action of the compound on the respective receptor. Previously, PPARγ agonists such as ciglitazone, mifepristone, 15d-PGJ2/15-PGJ(2), and pioglitazone were found to effectively alleviate cerebral ischemia-reperfusion injury in rats after MCAO, i.e., middle carotid artery occlusion [20,21,22] and bilateral common carotid artery occlusion [23]. Only a few studies have shown the neuroprotective potential of PPARγ agonists in post-treatment paradigms. These include thiazolidinediones such as rosiglitazone, which decrease the infarct volume and neurological deficits in rodents when applied until 6 h of reperfusion after MCAO [24,25]. Pioglitazone was found to evoke neuroprotection in terms of reduced brain infarction and neurological deficits when applied 2–5 days after MCAO [26]. Unfortunately, thiazolidinediones appeared hepatotoxic, which is why their clinical use is controversial. Moreover, newer thiazolidinediones, i.e., rosiglitazone and pioglitazone, have been linked to increased heart failure [27].

Compared to PPARγ agonists, selective PPARγ modulators such as amorfrutin B have safer pharmacological profiles and exhibit partial agonist or partial antagonist properties [12]. However, the neuroprotective capacity of selective PPARγ modulators in cellular and animal models of stroke is much less recognized than that of PPARγ agonists. The neuroprotection against hypoxia and ischemia attributed to selective PPARγ modulators is almost exclusively assessed on an example of telmisartan, which binds to the receptor in a different way than thiazolidinediones. Telmisartan, which was used as a pretreatment, suppressed cerebral injury in a murine model of transient focal ischemia but appeared ineffective in reducing stroke volume due to permanent ischemia [28]. The effect of pretreatment with telmisartan was reversed by antagonizing PPARγ with GW9662, thus confirming the agonizing action of the receptor modulator [29].

In the present study, we have provided evidence on the neuroprotective effect of post-treatment with amorfrutin B, the compound with proven properties of a selective PPARγ modulator [14,15]. The effect of amorfrutin B was estimated in terms of LDH, MTT, ROS activity, ROS-related 8-OHdG, and FJ-C, which suggests that amorfrutin B promotes mitochondrial integrity and is capable of inhibiting ROS activity and ROS-mediated DNA damage to prevent hypoxia- and ischemia-induced neural degeneration. Mitochondrial dysfunction is a major feature of degenerating neurons, and this phenomenon is connected with oxidative stress and PPARγ signaling. Recently, Xia et al. (2018) demonstrated that pretreatment with pioglitazone significantly alleviated ROS generation in cellular and animal models of cerebral ischemia, which is in line with the PPARγ-mediated neuroprotection evoked by amorfrutin B in our experiments [30]. A population study showed that blood concentrations of 8-OHdG are higher in patients with cerebral infarction than in healthy subjects [31]. In our study, this parameter was inhibited by a 6 h delayed post-treatment with amorfrutin B, as evidenced by the reduced level of 8-OHdG, which positions the compound among the most promising anti-stroke and wide-window therapeutics.

In our studies, hypoxia and ischemia evoked hypomethylation of global DNA. Similar to our studies, using next generation sequencing, Meller et al. [32] also showed that global DNA hypomethylation predominates as a response to preconditioning ischemia/harmful ischemia in the primary neuronal cultures subjected to oxygen and glucose deprivation. Although amorfrutin B did not affect hypoxia- and ischemia-evoked hypomethylation of global DNA in our study, it stimulated the methylation of the *Pparg* gene under hypoxic and ischemic conditions. Furthermore, amorfrutin B increased the protein level of PPARγ during hypoxia but decreased the mRNA and protein levels of PPARγ during ischemia, as evidenced by qPCR, Western blot, ELISA, and confocal microscopy. The expression of *Pparg*/PPARγ has been shown to depend on the duration of the injury and reoxygenation/reperfusion, as well as *Hif1a*/HIF-1α, which is known to activate the expression of PPARγ in response to hypoxia [33]. In our experimental models, the expression of *Pparg*/PPARγ was upregulated by hypoxia and ischemia, except for the protein level of PPARγ, which was not changed by hypoxia. Because amorfrutin B strongly inhibits *Hif1a* expression during hypoxia, we suggest that the amorfrutin B-evoked increase in the protein level of PPARγ during hypoxia does not involve a stimulatory effect of HIF-1α. However, the involvement of HIF-1α in the amorfrutin B-evoked decrease in PPARγ expression during ischemia cannot be excluded as amorfrutin B does not affect this factor during ischemia. It should also be stressed that, intriguingly, in the neurons of the ischemic hemisphere, despite increased PPARγ expression, PPARγ DNA binding activity is decreased, thus suggesting reduced transcriptional activity of the receptor [34,35]. This could explain our results, where no correlation was found between PPARγ expression and the expression of PPARγ-regulated factors, i.e., *Pgc1a*/PGC1α and *Adipoq*/ADIPOQ. In our study, the decrease in *Pgc1a* has occurred only in respect to mRNA expression level, but the level of PGC1α protein remained unchanged. The downregulation of *Pgc1a* gene may be related to complex and still not fully recognized role of sirtuins activity in PGC1α expression. Sirtuins activity is one of the possible way how the transcriptional pathway of *Pgc1a* can be regulated because sirtuins (SIRT1) are able to form a negative-feedback loop with PPARγ-related pathway [36]. In our studies, the sirtuins activity decreased after the treatment with amorfrutin B in hypoxic condition. We can speculate that this process initiated a negative-feedback loop with *Pgc1a* mRNA expression. What is more, RNA material to qPCR analyses has been collected after 18 h of reoxygenation; therefore, the observed changes in the expression level of *Pgc1a* may be secondary.

We demonstrated that under ischemic conditions, amorfrutin B-evoked hypermethylation of the *Pparg* gene was in line with the amorfrutin B-evoked decrease in the mRNA and protein expression of PPARγ. However, under hypoxic conditions, the amorfrutin B-dependent hypermethylation of the *Pparg* gene does not explain the amorfrutin B-dependent increase in receptor protein expression, suggesting other regulatory mechanisms. Even though hypermethylation is mostly related to a decrease in gene expression levels, recent studies shown that DNA hypermethylation may also be associated with upregulated gene expression [37,38]. We also hypothesize that this might be related to sirtuins because amorfrutin B normalized sirtuins activity in neuronal cells undergoing hypoxia but not ischemia. Sirtuins are known to be involved in various cellular processes, including hypoxic and ischemic injury; however, their specific roles are only partially recognized. For example, *Sirt3* knockout male mice are less vulnerable to ischemia/reperfusion or stroke injury [39], and upregulation of SIRT3 inhibits apoptosis in ischemia-challenged PC12 cells [40]. Intriguingly, in patients with acute cerebrovascular stroke, blood levels of SIRT1 are lower than those in control volunteers [41]. We postulate that hypoxia affects the specific set of sirtuins that differs from that affected by ischemia, and this specific set of sirtuins is vulnerable to post-treatment with amorfrutin B.

Regarding other epigenetic parameters, amorfrutin B did not change HDAC activity but stimulated HAT activity, both in hypoxic and ischemic conditions, even far above the level that has been detected in mouse neurons during normoxia. Enhanced HAT activity has been observed during adipogenesis when PPARγ expression was epigenetically stimulated [42]. Recent studies showed that change in histone acetylation homeostasis is common feature in neurodegenerative diseases including stroke. Both acetylation and deacetylation represent interesting targets for potential therapies in the treatment of hypoxic and ischemic diseases. In 2013, Lanzillotta et al. [43] using OGD model in primary cultures of mouse cortical neurons noticed that histone H3 acetylation was drastically decreased, while there were no change in HDAC activity that is similar to our observation in cell-based model of brain ischemia. According to the cited paper, these changes resulted from massive energy disturbances accompanying ischemia, reduction of pyruvate dehydrogenase activity and decreased generation of acetyl-CoA, an important cofactor for HAT activity. Therefore, we postulate that the increase in HAT activity after amorfrutin B post-treatment is the result of improving the cellular energy balance due to inhibition of oxidative stress and restoration of mitochondrial function. Furthermore, in our hypoxic model, amorfrutin B post-treatment reduced sirtuins activity which is known to be involved in epigenetic modifications by deacetylation. Previously, an inhibition of deacetylation was shown to partially prevent an impairment of histone acetylation [44] that is similar to the action of amorfrutin B in our study and suggests the HAT and sirtuins activities as potential therapeutic targets. According to our study, amorfrutin B appears to be a neuroprotective compound that prevents hypoxia- and ischemia-related epigenetic modifications, and in this way, it could recognize attractive targets that would serve to improve stroke pharmacotherapy.

## 5. Conclusions

In summary, 18 h post-treatment with amorfrutin B, which started at a 6 h delay from hypoxia and ischemia, evokes a strong neuroprotective effect against hypoxic and ischemic damage that involves PPARγ activation, hypermethylation of the *Pparg* gene, and dysregulation of *Pparg*/PPARγ expression, which would account for mitochondrial integrity maintenance and inhibition of oxidative stress and related DNA damage.

## Data Availability

The data that support the findings of this study are available from the corresponding author upon reasonable request. Some data may not be made available because of privacy or ethical restrictions.

## Supplementary Materials

The following are available online at https://www.mdpi.com/article/10.3390/biomedicines9080854/s1, Figure S1: Effects of amorfrutin B on caspase-3 activity, Table S1: Effects of amorfrutin B on studied parameters under the normoxic conditions.

## Abbreviations

8-OHdG	8-hydroxy-2′-deoxyguanosine
ADIPOQ/Adipoq	Adiponectin
Ct	Threshold cycle
DIV	Days in vitro
GW9662	2-Chloro-5-nitro-N-phenylbenzamide; irreversible PPARγ antagonist
HAT	Histone acetyltransferase
HDAC	Histone Deacetylase
HIF1α/*Hif1a*	hypoxia-inducible factor 1alpha
MAP2	Microtubule-associated protein 2
MCAO	Middle carotid artery occlusion
MTT	3-(4,5-dimethylthiazol-2-yl)-2,5-diphenyltetrazolium bromide
PGC1α/*Pgc1a*	Peroxisome proliferator-activated receptor gamma coactivator 1alpha
PPARγ/*Pparg*	Peroxisome proliferator-activated receptor gamma
rt-PA	Recombinant tissue plasminogen activator
RX	Retinoid X receptor
SPPARγMs	Selective PPARγ modulators

## References

[1] World Health Organization Stroke, Cerebrovascular Accident. https://www.emro.who.int/health-topics/stroke-cerebrovascular-accident/index.html.

[2] Frendl A., Csiba L. (2011). Pharmacological and Non-Pharmacological Recanalization Strategies in Acute Ischemic Stroke. Front. Neurol..

[3] Miller D.J., Simpson J.R., Silver B. (2011). Safety of Thrombolysis in Acute Ischemic Stroke: A Review of Complications, Risk Factors, and Newer Technologies. Neurohospitalist.

[4] Slowik A. (2014). New perspectives for acute stroke treatment: The role of mechanical thrombectomy. Adv. Interv. Cardiol..

[5] Kurinczuk J.J., White-Koning M., Badawi N. (2010). Epidemiology of neonatal encephalopathy and hypoxic–ischaemic encephalopathy. Early Hum. Dev..

[6] Zubčević S., Heljić S., Catibusić F., Užičanin S., Sadiković M., Krdzalic B. (2015). Neurodevelopmental Follow Up After Therapeutic Hypothermia for Perinatal Asphyxia. Med. Arch..

[7] DeRosa G., Sahebkar A., Maffioli P. (2018). The role of various peroxisome proliferator-activated receptors and their ligands in clinical practice. J. Cell. Physiol..

[8] Villapol S. (2018). Roles of Peroxisome Proliferator-Activated Receptor Gamma on Brain and Peripheral Inflammation. Cell. Mol. Neurobiol..

[9] Kernan W.N., Viscoli C.M., Furie K.L., Young L.H., Inzucchi S.E., Gorman M., Guarino P.D., Lovejoy A.M., Peduzzi P.N., Conwit R. (2016). Pioglitazone after Ischemic Stroke or Transient Ischemic Attack. N. Engl. J. Med..

[10] Cardoso S., Moreira P.I. (2020). Antidiabetic drugs for Alzheimer’s and Parkinson’s diseases: Repurposing insulin, metformin, and thiazolidinediones. Int. Rev. Neurobiol..

[11] Nanjan M., Mohammed M., Kumar B.P., Chandrasekar M. (2018). Thiazolidinediones as antidiabetic agents: A critical review. Bioorg. Chem..

[12] Chen Y., Ma H., Zhu D., Zhao G., Wang L., Fu X., Chen W. (2017). Discovery of Novel Insulin Sensitizers: Promising Approaches and Targets. PPAR Res..

[13] Weidner C., Wowro S.J., Freiwald A., Kawamoto K., Witzke A., Kliem M., Siems K., Müller-Kuhrt L., Schroeder F.C., Sauer S. (2013). Amorfrutin B is an efficient natural peroxisome proliferator-activated receptor gamma (PPARγ) agonist with potent glucose-lowering properties. Diabetologia.

[14] Lavecchia A., Di Giovanni C. (2015). Amorfrutins are efficient modulators of peroxisome proliferator-activated receptor gamma (PPARγ) with potent antidiabetic and anticancer properties: A patent evaluation of WO2014177593 A1. Expert Opin. Ther. Patents.

[15] Bin Samad M., Hasan N., Banarjee S., Rahman M., Raihan S., Banti F.L., Sayfe S.S., Hasan S.N., Akhter F., Kabir A.U. (2017). PEG modification of Amorfrutin B from Amorpha fructicosa increases gastric absorption, circulation half-life and glucose uptake by T3T-L1 adipocytes. Biomed. Pharmacother..

[16] Kajta M., Trotter A., Lasoń W., Beyer C. (2005). Effect of NMDA on staurosporine-induced activation of caspase-3 and LDH release in mouse neocortical and hippocampal cells. Dev. Brain Res..

[17] Kajta M., Lasoń W., Kupiec T. (2004). Effects of estrone on N-methyl-D-aspartic acid- and staurosporine-induced changes in caspase-3-like protease activity and lactate dehydrogenase-release: Time- and tissue-dependent effects in neuronal primary cultures. Neuroscience.

[18] Gu Q., Lantz S., Rosas-Hernández H., Cuevas E., Ali S.F., Paule M.G., Sarkar S., Lantz-McPeak S. (2014). In vitro detection of cytotoxicity using FluoroJade-C. Toxicol. Vitro.

[19] Wnuk A., Rzemieniec J., Lason W., Krzeptowski W., Kajta M. (2018). Apoptosis Induced by the UV Filter Benzophenone-3 in Mouse Neuronal Cells Is Mediated via Attenuation of Erα/Pparγ and Stimulation of Erβ/Gpr30 Signaling. Mol. Neurobiol..

[20] Pei L., Zhang Y., Zhang Y., Chu X., Zhang J., Wang R., Liu M., Zhu X., Yu W. (2010). Peroxisome proliferator-activated receptor γ promotes neuroprotection by modulating cyclic D1 expression after focal cerebral ischemia. Can. J. Physiol. Pharmacol..

[21] Wu J.-S., Tsai H.-D., Cheung W.-M., Hsu C.Y., Lin T.-N. (2015). PPAR-γ Ameliorates Neuronal Apoptosis and Ischemic Brain Injury via Suppressing NF-κB-Driven p22phox Transcription. Mol. Neurobiol..

[22] Wu X.J., Sun X.H., Wang S.W., Chen J.L., Bi Y.H., Jiang D.X. (2018). Mifepristone alleviates cerebral ische-mia-reperfusion injury in rats by stimulating PPAR γ. Eur. Rev. Med. Pharmacol. Sci..

[23] Shehata A.H., Ahmed A.-S.F., Abdelrehim A.B., Heeba G.H. (2020). The impact of single and combined PPAR-α and PPAR-γ activation on the neurological outcomes following cerebral ischemia reperfusion. Life Sci..

[24] Tureyen K., Kapadia R., Bowen K.K., Satriotomo I., Liang J., Feinstein D.L., Vemuganti R. (2006). Peroxisome proliferator-activated receptor-γ agonists induce neuroprotection following transient focal ischemia in normotensive, normoglycemic as well as hypertensive and type-2 diabetic rodents. J. Neurochem..

[25] Verma R., Mishra V., Gupta K., Sasmal D., Raghubir R. (2011). Neuroprotection by rosiglitazone in transient focal cerebral ischemia might not be mediated by glutamate transporter-1#. J. Neurosci. Res..

[26] Yu S.-J., Reiner D., Shen H., Wu K.-J., Liu Q.-R., Wang Y. (2015). Time-Dependent Protection of CB2 Receptor Agonist in Stroke. PLoS ONE.

[27] (2012). LiverTox: Clinical and Research Information on Drug-Induced Liver Injury.

[28] Kasahara Y., Taguchi A., Uno H., Nakano A., Nakagomi T., Hirose H., Stern D.M., Matsuyama T. (2010). Telmisartan suppresses cerebral injury in a murine model of transient focal ischemia. Brain Res..

[29] Li Y.-Y., Guo J.-H., Liu Y.-Q., Dong J.-H., Zhu C.-H. (2020). PPARγ Activation-Mediated Egr-1 Inhibition Benefits Against Brain Injury in an Experimental Ischaemic Stroke Model. J. Stroke Cerebrovasc. Dis..

[30] Xia P., Pan Y., Zhang F., Wang N., Wang E., Guo Q., Ye Z. (2018). Pioglitazone Confers Neuroprotection Against Ischemia-Induced Pyroptosis due to its Inhibitory Effects on HMGB-1/RAGE and Rac1/ROS Pathway by Activating PPAR-ɤ. Cell. Physiol. Biochem..

[31] Lorente L., Martín M., González-Rivero A., Pérez-Cejas A., Abreu-González P., Ramos L., Argueso M., Cáceres J., Solé-Violán J., Alvarez-Castillo A. (2021). DNA and RNA oxidative damage are associated to mortality in patients with cerebral infarction. Med. Intensiv..

[32] Meller R., Pearson A.N., Simon R.P. (2015). Dynamic Changes in DNA Methylation in Ischemic Tolerance. Front. Neurol..

[33] Zhao Y.-Z., Liu X.-L., Shen G., Ma Y.-N., Zhang F.-L., Chen M.-T., Zhao H.-L., Yu J., Zhang J.-W. (2014). Hypoxia induces peroxisome proliferator-activated receptor γ expression via HIF-1-dependent mechanisms in HepG2 cell line. Arch. Biochem. Biophys..

[34] Culman J., Zhao Y., Gohlke P., Herdegen T. (2007). PPAR-γ: Therapeutic target for ischemic stroke. Trends Pharmacol. Sci..

[35] Cai W., Yang T., Liu H., Han L., Zhang K., Hu X., Zhang X., Yin K.-J., Gao Y., Bennett M.V. (2018). Peroxisome proliferator-activated receptor γ (PPARγ): A master gatekeeper in CNS injury and repair. Prog. Neurobiol..

[36] Buler M., Andersson U., Hakkola J. (2016). Who watches the watchmen? Regulation of the expression and activity of sirtuins. FASEB J..

[37] Wan J., Oliver V.F., Wang G., Zhu H., Zack D.J., Merbs S.L., Qian J. (2015). Characterization of tissue-specific differential DNA methylation suggests distinct modes of positive and negative gene expression regulation. BMC Genom..

[38] Rauluseviciute I., Drabløs F., Rye M.B. (2020). DNA hypermethylation associated with upregulated gene expression in prostate cancer demonstrates the diversity of epigenetic regulation. BMC Med. Genom..

[39] Verma R., Ritzel R., Crapser J., Friedler B.D., McCullough L.D. (2019). Evaluation of the Neuroprotective Effect of Sirt3 in Experimental Stroke. Transl. Stroke Res..

[40] Li Y., Hu K., Liang M., Yan Q., Huang M., Jin L., Chen Y., Yang X., Li X. (2021). Stilbene glycoside upregulates SIRT3/AMPK to promotes neuronal mitochondrial autophagy and inhibit apoptosis in ischemic stroke. Adv. Clin. Exp. Med..

[41] Esmayel I.M., Hussein S., Gohar E.A., Ebian H.F., Mousa M.M. (2021). Plasma levels of sirtuin-1 in patients with cerebrovascular stroke. Neurol. Sci..

[42] Lee J.-E., Ge K. (2014). Transcriptional and epigenetic regulation of PPARγ expression during adipogenesis. Cell Biosci..

[43] Lanzillotta A., Pignataro G., Branca C., Cuomo O., Sarnico I., Benarese M., Annunziato L., Spano P., Pizzi M. (2013). Targeted acetylation of NF-kappaB/RelA and histones by epigenetic drugs reduces post-ischemic brain injury in mice with an extended therapeutic window. Neurobiol. Dis..

[44] Faraco G., Pancani T., Formentini L., Mascagni P., Fossati G., Leoni F., Moroni F., Chiarugi A. (2006). Pharmacological Inhibition of Histone Deacetylases by Suberoylanilide Hydroxamic Acid Specifically Alters Gene Expression and Reduces Ischemic Injury in the Mouse Brain. Mol. Pharmacol..

