# Supplementary material for: Post-Treatment with Amorfrutin B Evokes PPARγ-Mediated Neuroprotection against Hypoxia and Ischemia
Source: Biomedicines. 2021 Jul 21;9(8):854. doi: 10.3390/biomedicines9080854 (PMC8389580; doi:10.3390/biomedicines9080854)
Supplement: Supplementary file 1 [file biomedicines-09-00854-s001.zip › biomedicines-1279622-supplementary.pdf]

biomedicines

MDPI

Table S1. Effects of amorfrutin B on studied parameters under the normoxic conditions. Amorfrutin B (5 μM) posttreatment under normoxic conditions does not induce changes in MTT, Fluoro-Jade C, ROS activity and 8-OHdG level. Amorfrutin B does not induce alterations in the mRNA and protein expression levels, and immunofluorescence intensity except increase in *Pgc1a* mRNA level. Posttreatment with amorfrutin B does not affect epigenetic status of neuronal cells.

% of the control	MTT	Fluoro-Jade C	ROS Activity	Histone Acetyltransferase (HAT) Activity	PPARγ Intensity
normoxia	100 ± 2.819	100 ± 0.558	100 ± 2.793	100 ± 7.489	100 ± 7.372
normoxia + amorfrutin B	97 ± 2.755	102 ± 0.341	107 ± 2.458	102.936 ± 33.368	101.440 ± 7.303

pg/ml	8-OHdG
normoxia	199.538 ± 9.291
normoxia + amorfrutin B	165.202 ± 5.344

folds [*Hprt1* normalized]	*Hif1a*	*Pparg*	*Pgc1a*	*Adipoq*
normoxia	1.005 ± 0.051	1.002 ± 0.035	1.002 ± 0.032	1.044 ± 0.143
normoxia + amorfrutin B	1.012 ± 0.058	0.957 ± 0.063	1.245 ± 0.023*	1.519 ± 1.227

pg/μg of the protein	PPARγ	PGC1α	ADIPOQ
normoxia	0.0095 ± 0.0015	0.0020 ± 0.0007	2.29 ± 0.81
normoxia + amorfrutin B	0.0104 ± 0.0024	0.0020 ± 0.0005	2.04 ± 0.69

ng/μl	global DNA methylation
normoxia	14.755 ± 1.297
normoxia + amorfrutin B	13.199 ± 2.480

Methylation Rate	*Pparg* Gene Specific Methylation
normoxia	50.294 ± 2.408
normoxia + amorfrutin B	46.842 ± 4.817

μM/μg	histone deacetylase (HDAC) actvity
normoxia	1.414 ± 0.082
normoxia + amorfrutin B	1.527 ± 0.294

pM/μg	sirtuins activity	sirtuins activity + trichostatin A
normoxia	795.606 ± 23.013	283.678 ± 8.778
normoxia + amorfrutin B	745.230 ± 4.140	270.700 ± 0.860

Assessment of Caspase-3 Activity

After experiment neuronal cell cultures were lysed with the use of Caspase Assay Buffer. Then, cell lysates were treated with Ac-DEVD-pNA (N-acetyl-asp-glu-val-asp-p-nitro-anilide) which is a colorimetric substrate that is cleaved into p-nitroanilide in the presence of caspase-3. The levels of the obtained product were measured after 60 min with the use of Infinite M200PRO microplate reader (Tecan Mannedorf, Switzerland). Results were analyzed by i-control software and normalized to the absorbance of vehicle-treated cells. The data were presented as a percentage of the control ± SEM. The absorbances of blank samples were considered as no-enzyme controls and subtracted from each value.

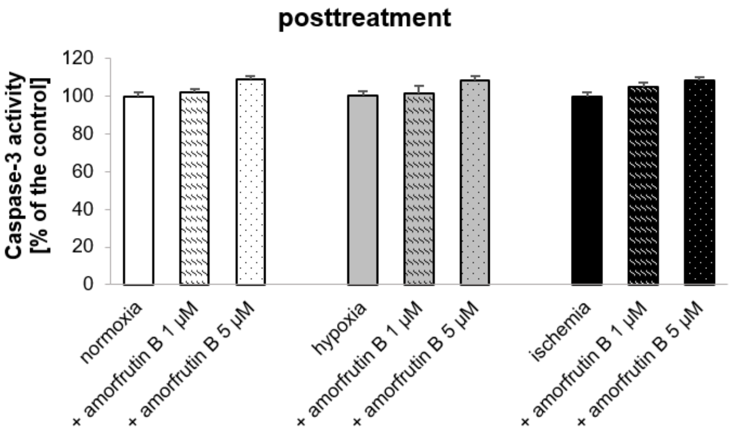

% of the control	**caspase-3 activity**
normoxia	100 ± 2.231
normoxia + amorfrutin B 1 µM	102 ± 1.664
normoxia + amorfrutin B 5 µM	109 ± 1.783
hypoxia	100 ± 2.642
hypoxia + amorfrutin B 1 µM	101 ± 4.182
hypoxia + amorfrutin B 5 µM	108 ± 2.761
ischemia	100 ± 1.730
ischemia + amorfrutin B 1 µM	105 ± 1.861
ischemia + amorfrutin B 5 µM	108 ± 1.576

Figure S1. Effects of amorfrutin B on caspase-3 activity**.** Hypoxic or ischemic conditions and exposure to amorfrutin B (1 and 5 µM) posttreatment did not contribute to changes in caspase-3 activity. The results are presented as a percentage of the control ± SEM of 3 independent experiments, consisting of 8-12 replicates per group.
